# Peptide Dimerization as a Strategy for the Development of Antileishmanial Compounds

**DOI:** 10.3390/molecules29215170

**Published:** 2024-10-31

**Authors:** Natália C. S. Coelho, Deivys L. F. Portuondo, Jhonatan Lima, Angela M. A. Velásquez, Valéria Valente, Iracilda Z. Carlos, Eduardo M. Cilli, Márcia A. S. Graminha

**Affiliations:** 1 Department of Clinical Analysis, School of Pharmaceutical Sciences, São Paulo State University (UNESP), Araraquara 14800-903, SP, Brazil; natalia.costa@unesp.br (N.C.S.C.); deivysleandro@gmail.com (D.L.F.P.); jhonatan.lima@unesp.br (J.L.); a.velasquez@unesp.br (A.M.A.V.); valeria.valente@unesp.br (V.V.); iracilda.zeppone@unesp.br (I.Z.C.); 2 Department of Biochemistry and Organic Chemistry, Institute of Chemistry, São Paulo State University (UNESP), Araraquara 14800-060, SP, Brazil

**Keywords:** dimerization, antimicrobial peptides, membrane, *Leishmania*, TSHa, temporin

## Abstract

Leishmaniasis is recognized as a serious public health problem in Brazil and around the world. The limited availability of drugs for treatment, added to the diversity of side effects and the emergence of resistant strains, shows the importance of research focused on the development of new molecules, thus contributing to treatments. Therefore, this work aimed to identify leishmanicidal compounds using a peptide dimerization strategy, as well as to understand their mechanisms of action. Herein, it was demonstrated that the dimerization of the peptide TSHa, (TSHa)_2_K, presented higher potency and selectivity than its monomeric form when evaluated against *Leishmania mexicana* and *Leishmania amazonensis*. Furthermore, these compounds are capable of inhibiting the parasite cysteine protease, an important target explored for the development of antileishmanial compounds, as well as to selectively interact with the parasite membranes, as demonstrated by flow cytometry, permeabilization, and fluorescence microscopy experiments. Based on this, the identified molecules are candidates for use in in vivo studies with animal models to combat leishmaniasis.

## 1. Introduction

Leishmaniasis (cutaneous, mucocutaneous, and visceral) is a serious global public health problem affecting 98 developing countries, including those in Latin America, East Africa, and the Mediterranean [1,2]. Annually, up to 1.2 million people are infected by parasites of the genus *Leishmania*, and 350 million are at risk of acquiring the disease [1,3]. These parasites have a series of biochemical differences that are important for the establishment of infection [4,5,6] and that can be explored as drug targets [7,8] since they are different from the counterpart vertebrates. One of these therapeutic targets in the parasite is the enzyme Cysteine protease, which the literature data indicate is essential for the survival and virulence of the parasite. Its inhibition may be used for the planning of new molecules [8,9,10]. The limited availability of drugs for the treatment of leishmaniasis, added to the diversity of side effects and the emergence of resistant strains, justifies the search for new antileishmanial compounds [11,12,13].

Antimicrobial peptides (AMPs) are among these substances, where the main mechanism of action is the disruption of membranes. In addition, they can present other mechanisms of action [14,15], including immune modulation in various organisms to protect them against the establishment of pathogens. The ability of AMPs to interact with cell membranes, permeating and disrupting them, is dependent on several factors, including the secondary structure, the total net charge (cationicity), the size, and the balance between the hydrophobic and hydrophilic regions [16]. AMPs have been studied for their antimicrobial, anticancer, antifungal, and antiparasitic activities [17], among others [16,18,19]. AMPs can have a variable number of amino acid residues (from 5 to 50) and are classified according to their secondary structure as β-sheet, α-helix, or unstructured [18]. Most AMPs have a disordered structure in an aqueous solution and assume an α-helical amphiphilic structure to facilitate interactions with the anionic lipid membrane. In contrast, β-sheet-structured peptides do not undergo a major conformational transition when interacting with the membrane due to the presence of stable disulfide bonds [15].

In parasites such as *Leishmania*, one of the main mechanisms of action of AMPs is on the cell membrane, formed mainly by negatively charged phospholipids, the main ones being phosphatidylinositol, phosphatidylserine, and phosphatidylethanolamine, which are anchored to glycocalyx [20]. Nonetheless, the morphology of the membrane varies according to the morphology of the parasite and its stage of development. This membrane is a selective therapeutic target, since the constitution of the cell membranes of mammals is different from that of the parasite, being less negative, enabling the selective effect of AMPs [21]. The initial step of action of an AMP is its interaction with the negatively charged parasite membrane, resulting in increased membrane permeability, leading to its lysis and the release of the cell contents. There are two important factors for this interaction with the membrane: the conformational structure of the peptide and the peptide-to-lipid ratio [15]. Despite the great potential of antimicrobial peptides, there are some challenges in the application of these molecules, such as potential toxicity and susceptibility to proteases, among others [22], which can be circumvented by the ability to chemically modify the AMP structure, thus altering its specificity and stability against proteases [23]. One of these strategies is peptide dimerization since peptide oligomerization also contributes to peptide activity, selectivity [24,25,26], and the resistant to proteases [27,28]. Dimeric peptides are mainly synthesized employing disulfide bonds, incorporating cysteine residues at any position in the peptide, as well as using branched Lys [27].

The class of AMP temporins can be highlighted for their broad spectrum of action against bacteria (Gram-positive and Gram-negative), fungi, and parasites [29,30]. Temporins are small molecules (13–14 amino acids) with a net positive charge ranging from 0 to +3 and an α-helix structure in membrane mimetics. They present a highly conserved N-terminal region that helps to anchor the peptide to cell membranes, together with a hypervariable and amidated C-terminal region, which may be involved in the selectivity and specificity of these molecules [31,32]. The temporin peptide TSHa (FLSGIVGMLGKLF-NH_2_) presents anti-*Leishmania infantum* activity [33], with IC_50_ of 18 μmol L^−1^. For this work, a temporin class peptide TSHa was dimerized based on a lysine residue in the C-terminus position.

The aims of this study were to synthesize and to characterize the dimeric peptide (TSHa)_2_K regarding its chemical nature and antileishmanial and cytotoxic activities, as well as to outline its possible mechanism of action.

## 2. Results and Discussion

### 2.1. Dimerization of the TSHa Peptide and Chemical Characterization

**Figure 1:**
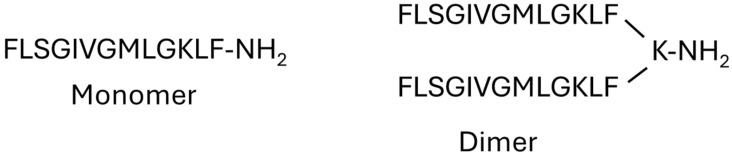
The structures of the monomeric TSHa and dimeric (TSHa)_2_K peptides.

One of the strategies used to enhance the microbial activity of AMPs is the dimerization of the peptides (Figure 1). According to the literature, dimerized peptides are less susceptible to degradation by proteases. This is due to the increase in interactions between the peptide chains in the dimer; at the same time, their biological properties are improved, such as potency and selectivity [27,34,35]. Dimerization improves the affinity of peptide chains for their specific targets. Furthermore, in pore-forming peptides, the oligomerization of the chains is important for their action, with dimeric peptides being more active due to the proximity between peptide chains.

**Figure 2:**
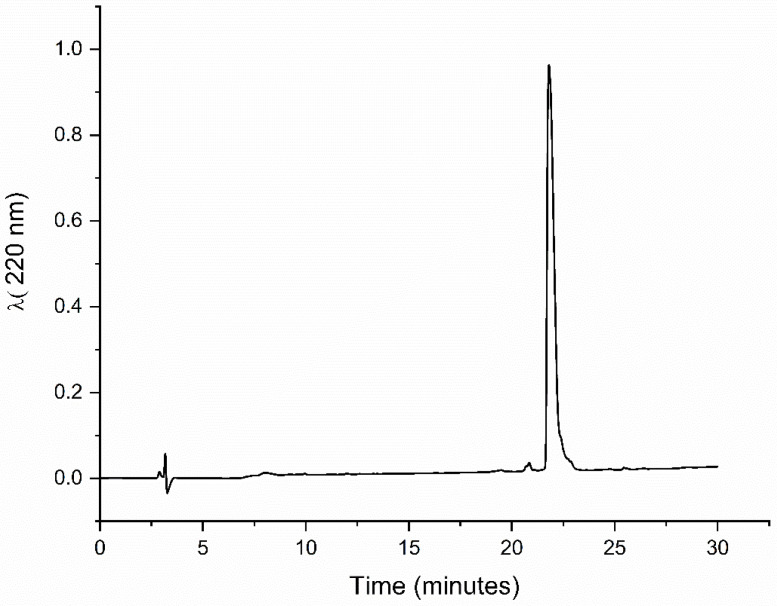
The chromatographic profile of (TSHa)_2_K, with a retention time of 21.8 min in a C18 column using a 5–95% gradient of solvent B in 30 min, an eluent flow rate of 1 mL min^−1^, and detection at 220 nm.

**Figure 3:**
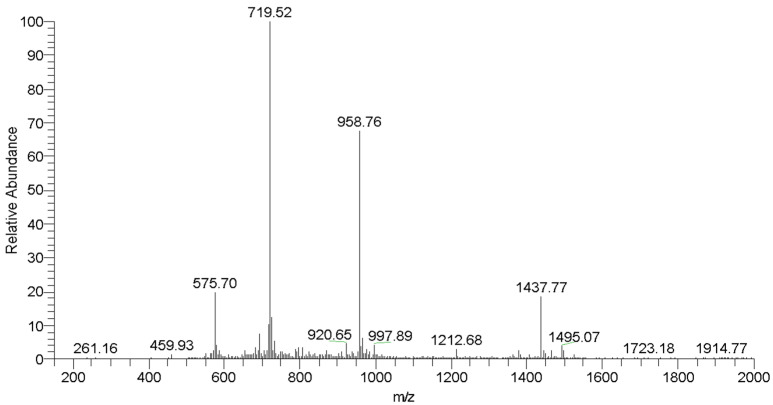
Mass spectrum of (TSHa)_2_K. Theorical MW: 2872 g mol^−1^. MW/Z obtained: 1437.8 (Z = 2), 958.8 (Z = 3), 719.5 (Z = 4), and 575.7 (Z = 5).

The obtained dimeric peptide was purified (Figure 2), and the molecular weight (MW) was confirmed by mass spectrometry (Figure 3).

### 2.2. Circular Dichroism

(TSHa)_2_K was evaluated by circular dichroism (CD) using a PBS buffer solution, LPC micelles, or a TFE solution. LPC is a surfactant that has a single hydrocarbon chain and can become organized in the form of micelles, which are spherical structures composed of lipophilic groups oriented towards the interior of the structure and hydrophilic groups oriented towards the outside, mimicking the environment of membranes. TFE is a structuring agent recognized as a secondary structure inducer.

**Figure 4:**
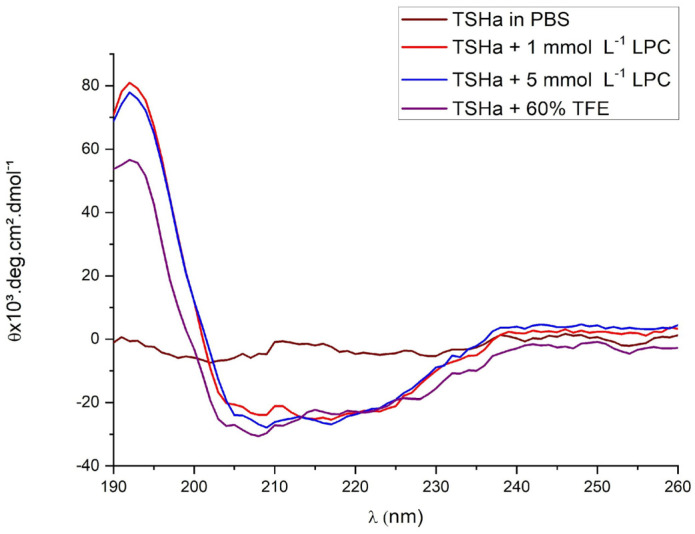
The CD spectra of the TSHa peptide in PBS solution, LPC at concentrations of 1 and 5 mmol L^−1^, and 60% TFE in PBS buffer. The peptide was evaluated at a concentration of 10 μmol L^−1^.

**Figure 5:**
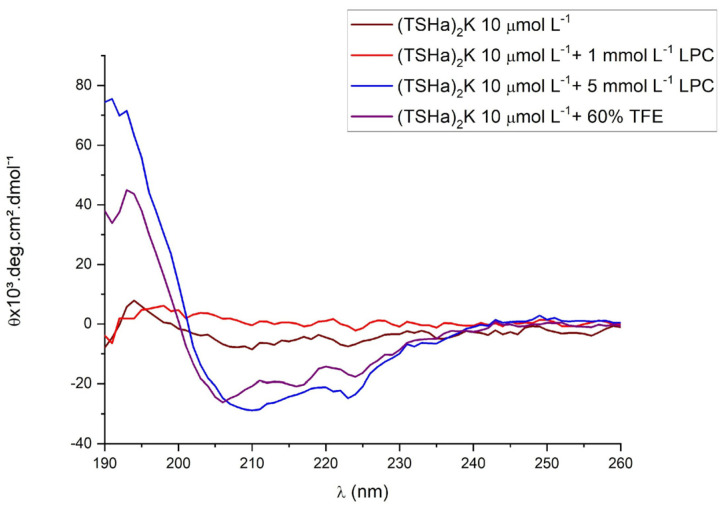
The CD spectra of the dimeric (TSHa)_2_K peptide in PBS solution, LPC at concentrations of 1 and 5 mmol L^−1^, and in 60% TFE in PBS buffer. The peptide was evaluated at a concentration of 10 μmol L^−1^.

The monomeric TSHa peptide in the PBS assay (Figure 4) exhibited a spectrum of disordered structures. In the presence of micelles or TFE, it was possible to observe a high incidence of α-helix structures [32,36] since this behavior is typical for AMPs, acting through a pore formation or carpet-like mechanism, causing membrane disruption. The disordered structure is modified by helical structures, as the first step of interaction with the membrane. For the dimeric (TSHa)_2_K peptide in the presence of PBS buffer and in 1 mmol L^−1^ LPC, it was possible to observe a non-ordered structure (Figure 5). At a concentration of 5 mmol L^−1^ LPC and in the presence of 60% TFE in PBS buffer, the spectra indicated a predominance of α-helix structures. The peptide acquired an increasing α-helical conformation, as shown by a positive band at 190 nm and negative bands at 208 and 222 nm. The difference in the CD spectra for the different concentrations of LPC could be attributed to the fact that the 1 mmol L^−1^ concentration was below the critical micellar concentration (CMC), whereas at 5 mmol L^−1^, the CMC had already been reached.

### 2.3. Biological Assays

#### 2.3.1. Biological Characterization: Leishmanicidal and Cytotoxic Activity Evaluation

The TSHa peptide has been shown to present antiparasitic activity against *L. amazonensis*, *L. infantum*, *T. cruzi*, and *T. brucei* [37,38,39,40]. In this work, TSHa showed significant leishmanicidal activity against *L. mexicana* promastigotes and amastigotes, with IC_50_ values of 6.3 and 8.1 μmol L^−1^, respectively, which were lower than the values of 18.1 and 22.8 μmol L^−1^ reported for *L. infantum* [41]. The dimeric peptide was almost 9 times more selective and 10× times more potent than the monomer against both the promastigote (IC_50_—0.6 μmol L^−1^) and amastigote forms (IC_50_—0.58 μmol L^−1^) of the parasite (Table 1). This demonstrated that the dimerization increased the potency and safety of these compounds (Table 1). Dimeric peptides composed of residues with a partial positive charge tend to interact more with anionic or zwitterionic lipid membranes, which can lead to the formation of pores, which may corroborate this high selectivity value of the peptide (TSHa)_2_K [32]. The dimeric (TSHa)_2_K peptide presented lower cytotoxicity and better selectivity (Table 1), with SI > 1724, which was approximately 45 times higher than that for the control drug Amp B (SI = 38), while the biological potencies were similar. Amp B is used for the treatment of several infections, including cutaneous leishmaniasis, but this molecule has side effects and toxicity [42]. Amp B can cause nausea, vomiting, chills, fever, hypertension, hypotension, and hypoxia, in addition to a high rate of nephrotoxicity. The clinical manifestations of Amp B nephrotoxicity include renal failure, hypokalemia, hypomagnesemia, metabolic academia, and polyuria due to nephrogenic diabetes insipidus [42].

Even when the amastigote treatment was extended to 48 h, all the tested peptides maintained their biological activity, with high selectivity and potency for the parasite, highlighting the dimeric peptide with IC_50_ = 0.47 μmol L^−1^ and SI > 2217 for the amastigote form (Table 1). The data were consistent with the literature, demonstrating that the dimerization strategy can increase the stability of peptides against proteases and also contribute to increasing the potency and selectivity of peptides.

Several models have been proposed for the mechanism of action of AMPs, with the main one being membrane permeabilization. The first step towards the disintegration of the membrane by amphipathic helical peptides, as found for the monomer and dimer of TSHa from the CD data, is the generation of a helical structure and interaction with the polar head groups of the membrane lipids. The positive charge of the peptide results in selective interaction with the negatively charged phospholipid monolayers of the *Leishmania* membrane. The main lipids of the promastigote membrane are neutral lipids, but they have a thick coating of lipophosphoglycans with a negative charge on the surface (glycocalyx). In addition, the amastigotes are surrounded by an endocytic vacuole of the host macrophage and have a high surface charge [43]. After insertion into the membrane, the pore (toroidal or barrel) formation or carpet-like mechanism occurs, destabilizing the membrane integrity [44]. Eukaryotic cells are generally neutral or have a small negative charge in the membrane, with AMPs being weakly attracted to this membrane composition (mainly phosphatidylcholine, phosphatidylethanolamine, sphingomyelin, and cholesterol).

**Figure 6:**
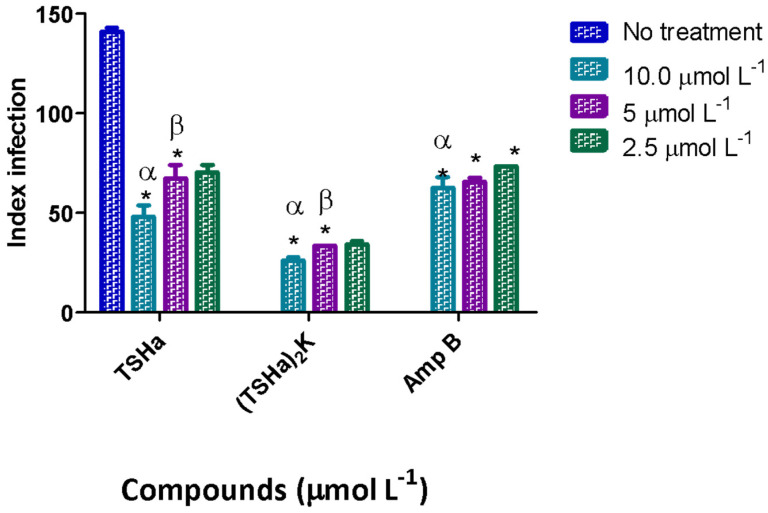
The infection indices for TSHa, (TSHa)_2_K, and Amp B in the intracellular amastigotes of *L. mexicana*. The infection index was calculated after 24 h of treatment. The negative control was untreated *L. mexicana* intracellular amastigotes. The data are expressed as the mean and standard deviation (SD) for 3 independent experiments. Two-way ANOVA (*p* < 0.05) was applied, where * indicates a significant difference between the tested compounds and the untreated control, and the different Greek letters indicate a significant difference between the tested concentrations of each compound.

**Figure 7:**
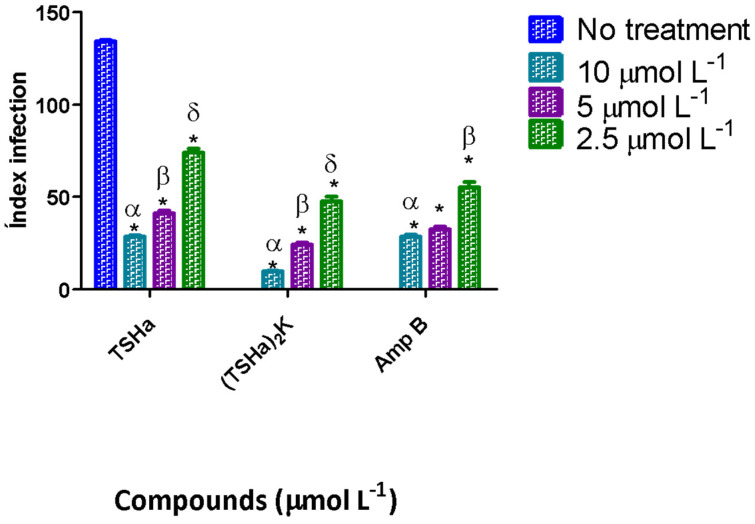
The infection indices for TSHa, (TSHa)_2_K, and Amp B in the intracellular amastigotes of *L. mexicana*. The infection index was calculated after 48 h of treatment. The negative control was untreated *L. mexicana* intracellular amastigotes. The data are expressed as the mean and standard deviation (SD) for 3 independent experiments. Two-way ANOVA (*p* < 0.05) was applied, where * indicates a significant difference between the tested compounds and the untreated control, and the different Greek letters indicate a significant difference between the tested concentrations of each compound.

During the infection process, the amastigotes replicated in macrophages are transmitted to healthy cells, leading to the amplification of infection [45]; the greater the parasitic load is, the worse the infection is. The infection index (percentage of infected cells x number of parasites per infected cell) was used to evaluate this transmission. In this work, the infection index was determined using *L. mexicana* for 24 h (Figure 6) and 48 h of treatment (Figure 7).

For the dimeric peptide, after 24 h, the infection rate decreased 5.5-fold compared to that of the infected cells that were not treated. In comparison to the control drug Amp B, the dimeric peptide was two times more effective in decreasing the infection rate, while the monomeric peptide showed similar values between the concentrations tested. After 48 h, the dimeric peptide and Amp B showed similar results, while the TSHa peptide showed a smaller decrease in the infection rate, indicating the possible degradation of this peptide after 48 h. In another study, the antimicrobial peptide filoseptin-1 (PS1) decreased the rate of infection in *L. amazonensis*, with the effect on amastigotes being concentration-dependent and the infection rate increasing with the concentration tested [46]. In this work, such concentration dependence was not observed, indicating a different mechanism of action. It is possible that the effects of the monomer and dimer peptides on the amastigotes occurred within the parasitophorous vacuoles and that the peptide interacted with the cell membranes of the macrophages, and subsequently with the cell membrane of the parasite. This interaction could lead to alterations in the flows of sodium and potassium, with a consequent increase in cell volume and the rupture of the parasite cell [46,47]. Another mechanism that might explain this decrease in the infection rate could be the interference of the peptides in the metabolic pathways of the parasite. For example, three magainin analogs (MG-H1, MG-H2, and MG-F5) led to dose-dependent decreases in ATP production in cultures of *Leishmania donovani*, causing parasite death [48].

**Figure 8:**
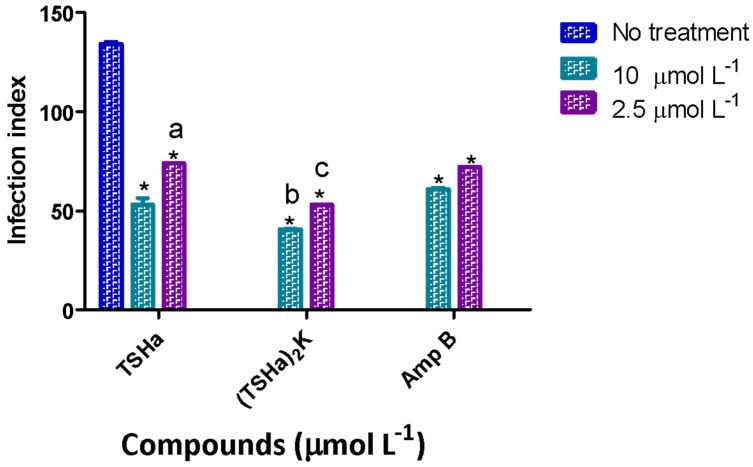
The infection indices for TSHa, (TSHa)_2_K, and Amp B in the intracellular amastigotes of *L. amazonensis*. The infection index was calculated after 24 h of treatment. The negative control was untreated *L. amazonensis* intracellular amastigotes. The data are expressed as mean and standard deviation (SD) for 3 independent experiments. Two-way ANOVA (*p* < 0.05) was applied, where * indicates a significant difference between the tested compounds and the untreated control, and the different letters indicate a significant difference between the tested concentrations of each compound.

The dimeric peptide was also evaluated against *L. amazonensis* (Table 2). The results were similar to those obtained in *L. mexicana*, with IC_50_ values of 0.6 and 0.47 μmol L^−1^ for the promastigote and amastigote forms, respectively. These data were indicative of the activity of (TSHa)_2_K against the different *Leishmania* species. The effect of the (TSHa)_2_K dimer on the infection rate was also evaluated, revealing a 3.8-fold decrease in the infection rate compared to that of the infected cells that were not treated, with the effect being greater than that observed for Amp B (Figure 8).

There are various factors that may affect the infection process and virulence of *Leishmania*, such as the strain or species of the parasite and immunological factors, such as cysteine proteases (CPs). *Leishmania* expresses high levels of several classes of CP belonging to the papain family, which are crucial for parasite metabolism, reproduction, and intracellular survival [49,50]. During the *Leishmania* infection process in macrophages, CPB modulates the host responses by negatively regulating protective Th1 immune responses, particularly the production of IFN-γ, by the degradation of the transcription factor NF-κB, and the subsequent inhibition of production of IL-12 by infected macrophages. Therefore, the protozoan CPB can alter the signaling of host macrophages, increasing the transcription of IL-12, which makes the establishment of infection difficult [50,51,52]. The data reported in the literature, including the mechanism of action of peptides, have demonstrated the fundamental role of cysteine proteases of the parasite in the infectious process, suggesting the high therapeutic potential of inhibiting these enzymes [49,50]. It has been shown that CPB inhibitors can reduce the infectivity of *L. mexicana* both in vitro and in vivo, demonstrating that these are virulence factors [53]. CPB is more commonly expressed in amastigote form and plays an important role in the interaction between *Leishmania* and its mammalian host, in which the inhibition of this enzyme can decrease the infectivity of macrophages [54]. Hence, (TSHa)_2_K is expected to be more active against CPB, leading to a lower infection rate, compared to those of the monomer peptide and Amp B.

#### 2.3.2. Stability Assay with Active Fetal Bovine Serum and the Promastigote Form of *L. amazonensis*

There are numerous peptide candidates that can be used clinically, but the transition of AMPs from the laboratory to the market has been greatly hampered by their rapid degradation by plasma proteases and hepatic and renal enzymes [55]. Several strategies have been employed to increase the stability of AMPs, including the incorporation of non-natural amino acids, the modification of the N and/or C terminal, cyclization, the use of non-peptide structures (peptidomimetics), and the multimerization of AMP monomers [56,57]. Therefore, stability studies were carried out with these molecules in the presence of the *Leishmania* parasite in its promastigote form, with active fetal bovine serum added to the culture medium. This serum contains active proteins and proteases that could degrade the molecules, enabling the assessment of peptide stability and whether any degradation products could still have biological activity. Assays were performed using the TSHa and (TSHa)_2_K peptides. Although the leishmanicidal activity level was lower than the IC_50_ value of 8.0 μmol L^−1^ reported previously [58], the TSHa peptide presented significant leishmanicidal activity against *L. amazonensis* promastigotes, with an IC_50_ of 20 μmol L^−1^ (Table 3), even in the presence of proteases. The dimeric peptide showed an IC_50_ of 6.12 μmol L^−1^ (Table 3) for the promastigote form, demonstrating significant biological activity. Hence, although the compounds showed some type of degradation in the presence of the active serum, they nonetheless maintained relevant biological activity.

### 2.4. Study of Mechanism of Action

The physical–chemical properties revealed that the peptides investigated here have a positive charge, facilitating interactions with the parasite membranes by electrostatic forces. This is the first step towards the disruption of the membrane of the parasite or the parasitophorous vacuole and the permeation of the peptides through the membrane of the host cell [59]. The peptides may also redistribute membrane components such as sterols, form specific phospholipid microdomains, or directly interact with membrane proteins and alter their function [60]. To obtain information about the mechanism of action of the TSHa peptide and its dimeric analogue, concerning their interactions with biological membranes, analyses were performed using flow cytometry assays, confocal microscopy, and fluorescence microscopy. In addition, to identify whether one of the targets of these molecules is an intracellular target, the inhibition of an important virulence factor for the parasite, CPB, was investigated.

#### 2.4.1. Vesicle Permeabilization Assays

The liposomal Amp B formulation represents the best therapeutic drug used for the treatment of leishmaniasis; Amp B binds to ergosterol and creates pores in the parasite membrane, affecting cell permeability, leading to the loss of cations such as K^+^ and triggering the cell death process [61].

To evaluate the effect of monomeric and dimeric peptides on the lipid membrane and relate it to the mechanism of action, vesicle permeabilization studies were carried out for evaluating the possible mechanisms on membrane. The leakage assays were carried out with carboxyfluorescein (CF) internalized in the mimetic membranes. The vesicles of POPC–Chol (4:1) and POPC–POPS (phosphatidylserine)–ergosterol (16:3:1) were employed. These mimetic membranes were designed considering the compositions of phospholipids and steroids in the host membrane and in the Leishmania membrane [62,63], respectively. The constitution of the cell membrane of Leishmania varies according to the morphology of the parasite and its stage of development, but is more negative than the cell membranes of mammals, which allow for cationic AMPs to bind more easily [21]. Our studies were based on the membrane of the promastigote form, which is characterized by the presence of mainly phosphatidylcholine, phosphatidylethanolamine, phosphatidylinositol, phosphatidylserine, and ergosterol, in addition to diglycerides and glycolipids containing mannose and galactose [64]. In the amastigote form of Leishmania, there is a higher concentration of fatty acids and cholesterol, while triglycerides and ergosterol decrease during the transition from promastigotes to amastigotes. In addition, the main phospholipids found are sphingomyelin and phosphatidylserine, with lower proportions of phosphatidylinositol and lysophosphatidylethanolamine [65].

**Figure 9:**
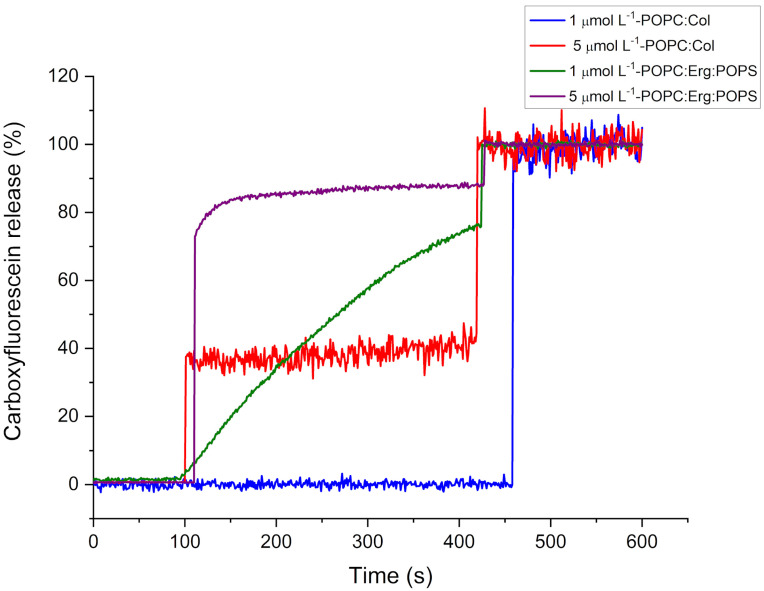
Carboxyfluorescein released by the TSHa peptide from the POPC–Chol (4:1) and POPC–POPS–Erg (16:3:1) vesicles.

The action of the TSHa peptide on the POPC–Chol vesicles, which mimic the host cell membrane (macrophages), at a concentration of 1 µmol L^−1^ did not release CF, while the use of a 5 µmol L^−1^ concentration resulted in the 38% release of CF. The permeabilization was greater in the POPC–POPS–Erg vesicles than that in the POPC–Chol vesicles, with around 70 and 85% CF release for concentrations of 1 and 5 µmol L^−1^, respectively, indicating higher selectivity for this more negative vesicle (Figure 9).

**Figure 10:**
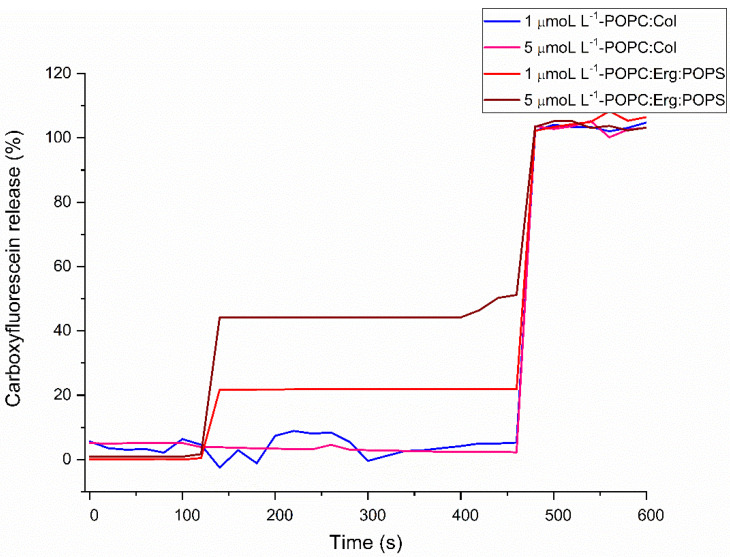
Carboxyfluorescein released by the (TSHa)_2_K peptide from the POPC–Chol (4:1) and POPC–POPS–Erg (16:3:1) vesicles.

The dimeric (TSHa)_2_K peptide was also able to permeabilize the POPC–POPS–Erg vesicles at concentrations of 1 and 5 µmol L^−1^, with CF release of 20 and 45%, respectively (Figure 10). Permeabilization was concentration-dependent, related to the mechanism of action of pore formation, with a higher amount of peptide resulting in a greater number of pores [66]. No permeabilization was found for the POPC–Chol vesicles, suggesting that the dimerization strategy increased the selectivity of this compound for vesicles that mimic the parasite membrane. Selectivity could be explained by the presence of negatively charged lipids in the vesicle mimicking the membrane of the parasite, allowing for the dimeric (TSHa)_2_K, which has a partial positive charge, to interact more strongly with the parasite membrane compared to that of the host. These lipids accumulated in the outer leaflet of the bilayer, and subsequently became organized into microdomains in the bilayer, affecting the permeability of the membrane, which led to the release of CF.

The permeabilization capacity of temporin peptides has been attributed to the α-helix structure that allows for interaction with the membrane, promoting its permeabilization by the formation of toroidal pores and channel aggregates. At high concentrations, these peptides can act according to the so-called carpet-like mechanism [31,67], which involves the dissolution of the membrane by the formation of micellar structures, with an action analogous to that of a detergent.

#### 2.4.2. Flow Cytometry Analyses

**Figure 11:**
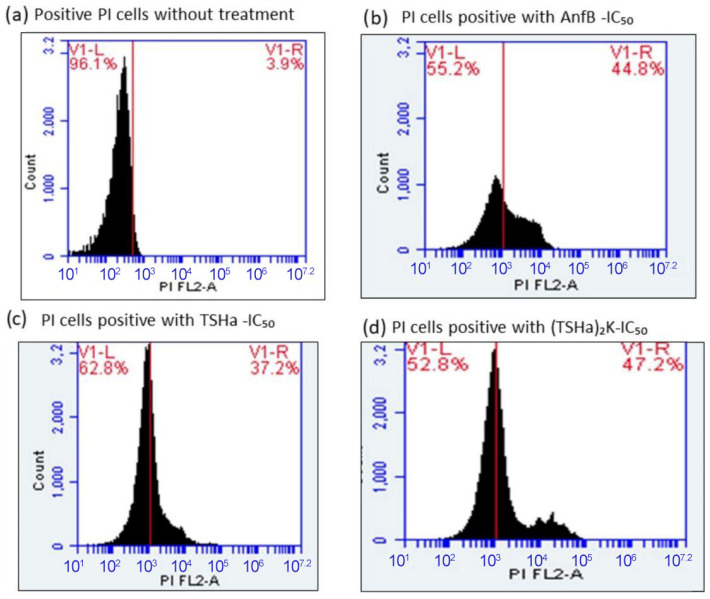
The flow cytometry membrane damage analysis of the promastigote form of *L. mexicana* at a concentration of 5 × 10^6^ cells mL^−1^ and labeled with PI (80 µg mL^−1^) for 20 min. (**a**) The parasites without PI treatment, (**b**) the parasites treated with Amp B at a concentration of 1 × IC_50_, (**c**) the parasites treated with TSHa at a concentration of 1 × IC_50_, and (**d**) the parasites treated with (TSHa)_2_K at a concentration of 1 × IC_50_.

The mechanism of action of the peptides on the membrane was also evaluated by flow cytometry using propidium iodide. Flow cytometry has been considered more reliable for studies of cell susceptibility because it provides intrinsic information about cells, such as size and complexity [68]. The use of fluorochromes allows for the evaluation of a wide range of physiological or morphological parameters, such as membrane integrity, pH, membrane potential, and viability. The results presented here (Figure 11) are expressed as the fluorescence intensity of the PI-labeled *L. mexicana* cells, considering that the damage caused to the parasite membrane facilitated the entry of PI into the cell nucleus.

**Figure 12:**
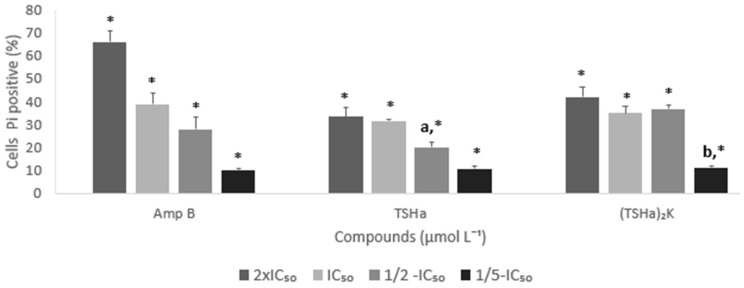
Percentages of the *L. mexicana* promastigotes marked with propidium iodide (PI) after the treatment with Amp B, TSHa, and (TSHa)_2_K at 2 × IC_50_, IC_50_, ½ × IC_50_, and ⅕ × IC_50_ for 24 h. All the data were submitted to statistical analysis (two-way ANOVA and Tukey’s test, with *p* < 0.05 indicating a significant difference between the compounds tested*, and p < 0.01 indicating a significant difference between the tested concentrations.* The results showed significant differences between the tested compounds (*), and the different letters indicate a significant difference between the tested concentrations of each compound.

In these assays, concentrations based on the IC_50_ in promastigote form of *L. mexicana* of each compound were adopted, with values of 2 × IC_50_, 1 × IC_50_, ½ × IC₅₀, and ⅕ × IC₅₀. The monomeric and dimeric peptides were able to cause damage to the membrane of the promastigote form of *L. mexicana*. At a concentration of 1 × IC_50_, approximately 38% of the parasite cells were labeled with PI (Figure 11c,d). The permeabilization of the parasite membranes was directly proportional to the concentration used (Figure 12), corroborating the hypothesis that the mechanism of action of the molecules was by the formation of pores in the membrane [69]. The data reported in the literature showed that the absence of LPG (lipophosphoglycan) and PPG (proteophosphoglycan) can contribute to the resistance of the promastigote form of *L. mexicana* [70]. Hence, the small size and low load of the temporins could facilitate diffusion from the glycocalyx to the plasma membrane.

#### 2.4.3. Study of the Mechanism of Action of the Peptides Using Fluorescence Microscopy

**Figure 13:**
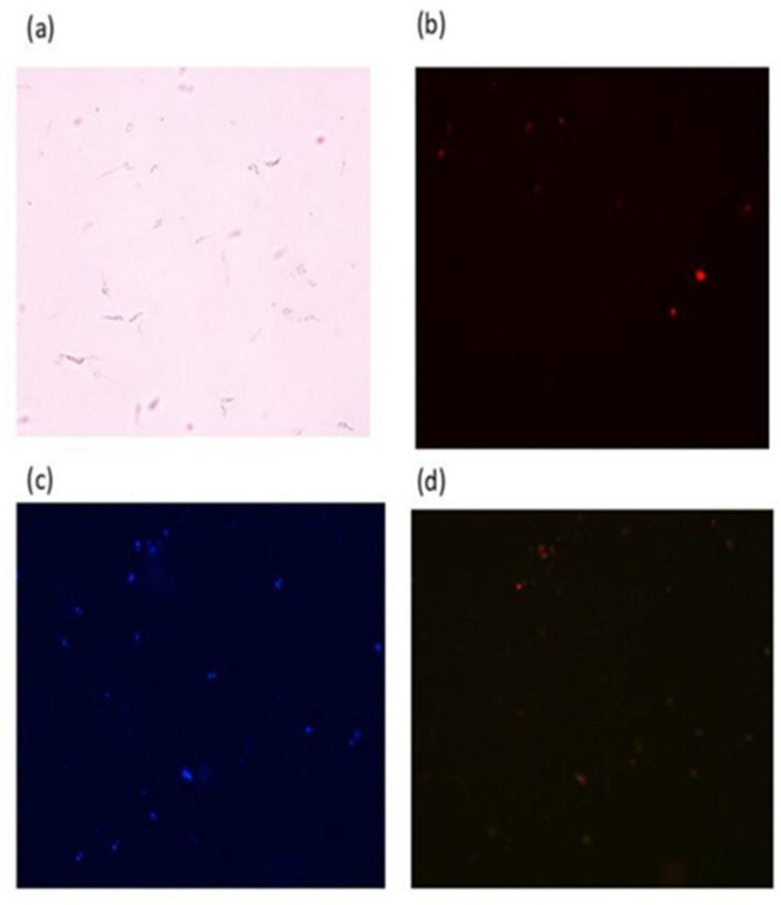

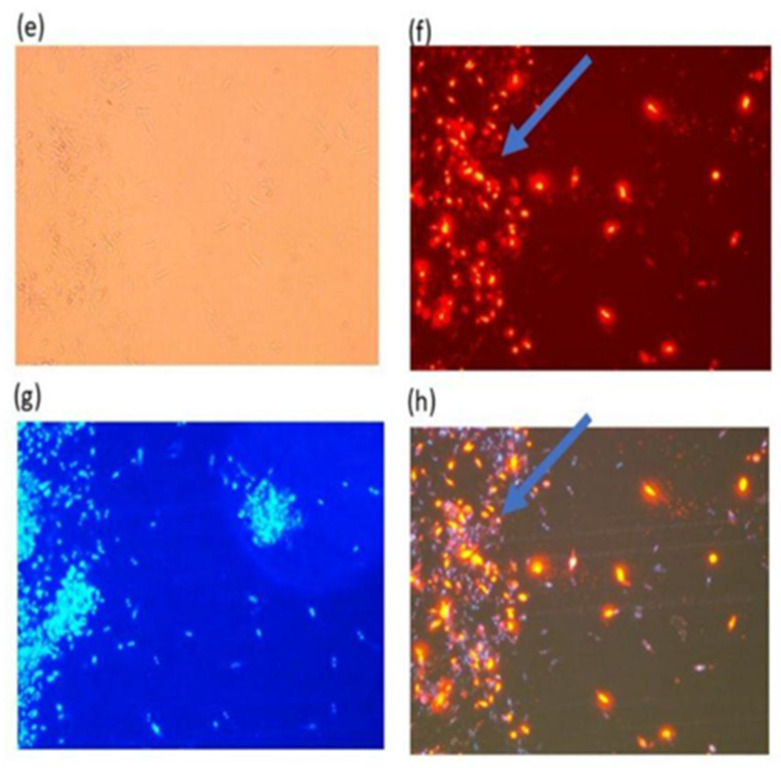
Fluorescence microscopy assay with promastigotes of *L. mexicana* at concentration of 1 × 10⁷ cells mL^−1^ without treatment for 24 h. (**a**) Cells without staining; (**b**) *L. mexicana* stained with 40 µg mL^−1^ PI for 5 min; (**c**) *L. mexicana* stained with 5 µg mL^−1^ Hoechst for 5 min; (**d**) merging of two stains. In (**e**–**h**), promastigotes of *L. mexicana* at concentration of 1 × 10⁷ cells mL^−1^ treated with 2.5 µmol L^−1^ of (TSHa)_2_K peptide for 24 h. (**e**) Cells without staining; (**f**) *L. mexicana* stained with 40 µg mL^−1^ propidium iodide for 5 min; (**g**) *L. mexicana* stained with 5 µg mL^−1^ Hoechst for 5 min; (**h**) merging of two stains. Arrows indicate example of conglomerates of parasites.

Fluorescence microscopy assays were also employed to investigate the mechanism of action of the peptides. The negative control cultures of *L. mexicana* without treatment (Figure 13a) were incubated with the different fluorophores, and we observed lots of staining by the Hoechst fluorophore (Figure 13c), indicating that the cells were viable, and less PI staining (Figure 13b). In addition, the ratio of PI-labeled cells to Hoechst-labeled cells was used to determine the percentage of cells that had suffered some type of membrane damage. Images were also obtained after the treatments of the promastigote form with the TSHa peptide, the dimeric (TSHa)_2_K peptide, and the Amp B control drug at concentrations of 2.5 and 1.25 µmol L^−1^ after 2 and 24 h to evaluate the mechanism of action of these molecules and whether the time of exposure to the compounds might affect their activity. Figure 13e–h shows the results using (TSHa)_2_K at 2.5 µmol L^−1^ for 24 h. The results for the other treatments are provided in the Supplementary Materials (Figures S1–S10).

**Figure 14:**
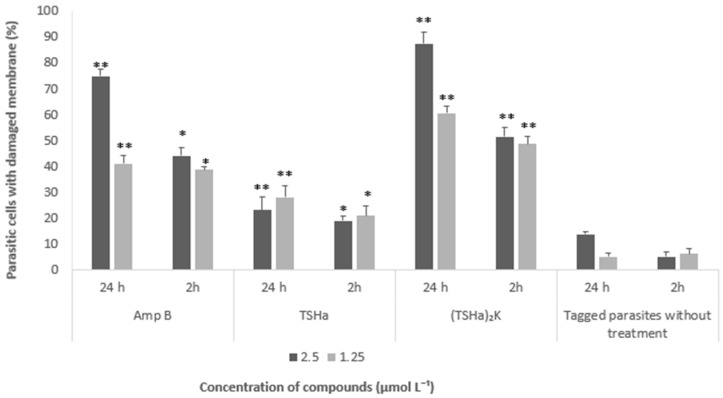
The percentages of *L. mexicana* promastigotes that suffered membrane damage calculated using the ratio of the results for parasites labeled with propidium iodide stain and parasites labeled with Hoechst stain after the treatments with Amp B, TSHa, and (TSHa)_2_K at concentrations of 1.25 and 2.5 µmol L^−1^ for 2 and 24 h. The data were subjected to statistical analysis using two-way ANOVA and Tukey’s test, with *p* < 0.05 indicating a significant difference between the tested compounds, and *p* < 0.01 indicating a significant difference between the tested concentrations. The results showed significant differences between the tested compounds (*) and between the tested concentrations of each compound (**).

The results corroborate that the mechanism of action is related to pore formation on the parasite membranes. In addition, microscopy analyses revealed the formation of conglomerates of parasites (arrows—Figure 13), many of which had lost their base form, which is indicative of the dissolution of the membrane [71]. This was also an indication that the parasites began the process of cell death, with the marking by PI becoming more evident [72]. Figure 14 shows the numbers of parasites that suffered damage under the different treatment conditions. The data obtained after 24 h confirmed that Amp B and (TSHa)_2_K caused damage to the membrane and that the damage was concentration-dependent, with a higher concentration of the compound causing greater damage. For both the compounds, a longer treatment time led to greater damage.

#### 2.4.4. Cysteine Protease (CPB) Enzyme Inhibition Assay

In addition to the membrane, peptides may have other therapeutic targets in *Leishmania*. Enzymes are essential for functional integrity and virulence, highlighting the fundamental role of cysteine proteases in the infectious process, so their inhibition can have high therapeutic potential, enabling the design of new drugs [8,51,54,73]. In *L. mexicana*, three cysteine protease genes were identified as virulence factors: lmcpa, lmcpb, and lmcpc. Of these, lmcpb encodes a protein product like cathepsin L and is the most abundant in the amastigote form of the parasite, so this enzyme is an important therapeutic target. Among its inhibitors, it has been shown that a derivative of benzo[b]thiophene ((4aS,9bS,9cS)-4-methyl-9c-(4-methylbenzoyl)-4a-phenyl-4,4a,9b,9c-tetrahydro-1H-benzo[4,5]thieno[2,3-b]furo[3,4-d]pyrrole-1,3(3aH)-dione) is a potent and selective inhibitor of LmCPB2.8△CTE (CEC), with an IC_50-CEC_ of 3.7 µmol L^−1^ [50]. Guanidinic compounds can also target the cysteine protease, highlighting the compound E64 (L-trans-epoxysuccinyl-leucylamido-(4-guanidino)butane), which in the nanomolar range is a potent irreversible inhibitor of *Leishmania* CPB [8,12,50]. Compounds belonging to the azapeptide class have been reported to be inhibitors of cysteine proteases, such as papain, cathepsin B, and cathepsin K. One of these compounds, Z-Arg-Leu-Val-Gly-Ile-Val-O-methyl is a potent inhibitor of cathepsin B, with a Ki value of 0.088 nmol L^−1^ [74], demonstrating the high potential of peptides as cysteine protease inhibitors.

Based on this, evaluation was conducted of the capacity of the peptides to inhibit CPB. The TSHa and dimer peptides both inhibited more than 90% of the enzyme activity, with the highest inhibition by the dimer peptide (Table 4). The IC_50_ values of the molecules against the CPB enzyme were determined (Table 4), with a lower IC_50_ of around 3.6 μmol L^−1^ for the dimeric (TSHa)_2_K peptide, highlighting its capacity as a CPB inhibitor.

The inhibition achieved with the dimeric peptide indicated that the mechanism of action of this molecule against *Leishmania* not only targeted the membrane, but also aided in the inhibition of cysteine protease, corroborating the lower IC_50_ against the amastigote and demonstrating the great potential of this molecule. The results were also consistent with the infection index data for the TSHa and (TSHa)_2_K peptides. These findings indicate the potential for the development of new candidate drugs for the treatment of leishmaniasis.

## 3. Materials and Methods

### 3.1. Peptide Synthesis

The peptides were synthesized using standard solid phase peptide synthesis. The TSHa peptide was synthesized using Rink Amide resin (Aapptec, Louisville, KY, USA), with a substitution degree of 0.5 mmol L^−1^. For the synthesis of the TSHa dimer, (TSHa)_2_K, an Fmoc-Lys(Fmoc) residue (Aapptec, Louisville, KY, USA) was used as a linker between the two TSHa chains. The lysine residue was coupled to 0.23 mmol g^−1^ substitution-grade PEG Rink Amide resin (Aapptec, Louisville, KY, USA). The decrease in the substitution degree was necessary to increase the yield of the synthesis. An α-amino group deprotection step was performed in 20% 4-methyl-piperidine (TCI, Miami, FL, USA)/dimethylformamide (DMF) (Êxodo Científica, Sumaré, SP, Brazil) for 1 and 20 min. The amino acids were coupled at two-fold excess using N,N’-diisopropylcarbodiimide (DIC)/N-hydroxybenzotriazole (HOBt) Aapptec, Louisville, KY, USA) in DMF. Cleavage was performed with 94% trifluoroacetic acid (Sigma-Aldrich, St. Louis, MO, USA), 1% TIS (triisopropylsilane) (Sigma-Aldrich, St. Louis, MO, USA), 2% ultrapure water and 2% EDODT (2,2′-(Ethylenedioxy)diethanethiol) (Sigma-Aldrich, St. Louis, MO, USA). Subsequently, the peptide and resin were precipitated with ice-cold ethyl ether, followed by discarding the supernatant. The peptides were extracted with 50% solvent A (H_2_O containing 0.045% TFA in solvent B (acetonitrile containing 0.036% TFA), and the resulting supernatants were lyophilized.

The resulting products were purified by semi-preparative HPLC employing a C18 reversed-phase column (Jupiter Proteo, Phenomenex, Milano, Italy, 25 cm × 10 mm, 5 μm particles). Elution was performed using solvent A and solvent B at a flow rate of 5 mL min^−1^, with detection at 220 nm. The purity of each fraction was determined using HPLC in analytical mode, with a C18 column (25 cm × 10 mm) and elution using a gradient from 5 to 95% solvent B over 30 min, with an eluent flow rate of 1 mL min^−1^. Detection was performed at 220 nm with a Shimadzu spectrometer. The molecular weights of the peptides were analyzed using a mass spectrometer in ESI-IT-MS configuration (LCQ Fleet, Thermo Fisher Scientific, Waltham, MA, USA), with an ion trap analyzer in positive electrospray mode (M + H)^+^ and a range of 200–2000 g mol^−1^.

### 3.2. Circular Dichroism

The peptides were structurally characterized at 10 µmol L^−1^ by circular dichroism (CD) spectroscopy in the range 195–260 nm using a JASCO J-815 CD spectrophotometer (Center for Macromolecular Interactions, Boston, MA, USA) with nitrogen flushing in 1 mm path length quartz cuvettes at 25 °C. The experiments were performed using LPC (lysophosphatidylcholine) at concentrations of 1 and 5 mmol L^−1^ in PBS (phosphate-buffered saline, pH 7.4) and in 60% 2,2,2-trifluoroethanol (TFE) structuring solution. The spectra were recorded as an average of five scans. The data were collected in millidegrees and were converted to molar ellipticity (θ, in deg cm^2^ dmol^−1^) [75].

### 3.3. Biological Assays

The *L. amazonensis* promastigotes (MPRO/BR/1972/M1841-LV-79) were grown in LIT culture medium, while the *L. mexicana* promastigotes (MNYC/BZ/62/M379) were cultivated in Schneider’s medium (Sigma-Aldrich, St. Louis, MO, USA), supplemented with 10% heat-inactivated fetal bovine serum (FBS; Gibco/Invitrogen, Waltham, MA, USA) [13] at 28 °C until reaching the mid-log phase of growth. Macrophages were sourced from the peritoneal cavity of Swiss mice using the methodology outlined by de Almeida et al. [76]. Anti-promastigote and anti-amastigote assays of *L. mexicana* and *L. amazonensis* and their cytotoxic activity were conducted as previously described [58]. IC_50_ is the concentration capable of killing 50% of the parasites. The drug concentration that inhibited 50% of cell growth was denoted as the 50% cytotoxic concentration (CC_50_). Cytotoxicity for the host cells and the protozoan species was compared and expressed as the selectivity index (SI), calculated as the ratio of the CC_50_ for macrophages to the IC_50_ for the protozoan species.

### 3.4. Serum Stability

Peptide stability was investigated using anti-promastigote assays (as described by Costa et al. 2023 [58]) with *L. amazonensis* and LIT culture media with fetal bovine serum.

### 3.5. Vesicle Preparation and Permeabilization Assay

Large unilamellar vesicles (LUVs) composed of POPC (1-palmitoyl-2-oleoyl-sn-glycero-3-phosphatidylcholine) and cholesterol (Chol) (4:1), or POPC, POPS (1-palmitoyl-2-oleoyl-sn-glycero-3-serine), and ergosterol (Erg) (16:3:1) were prepared according to the methods described by Lorenzón et al. [34]. The rate of release of CF from the vesicles was measured using fluorescence intensity at 495 and 517 nm after the addition of TSHa at 1 and 5 μmol L^−1^, (TSHa)_2_K at 1 and 5 μmol L^−1^, and the reference drug Amp B at 1 and 5 μmol L^−1^. We employed a Hitachi U-2000 spectrophotometer (TSHa) (Hitachi, Tokyo, Japan) or a Fluorolog-3 FL3-122 spectrofluorometer (Horiba Jobin Yvon, Glasgow, UK) ((TSHa)_2_K) for analyses.

### 3.6. Membrane Damage Assays Using Flow Cytometry

For flow cytometry assays, the promastigotes of *L. mexicana* were grown in Schneider medium (Sigma) supplemented with inactivated fetal bovine serum. The culture of the parasites was adjusted to a concentration of 5 × 10^6^ cells mL^−1^, followed by incubation in the presence or absence of the peptides or Amp B at concentrations of 2 × IC_50_, 1 × IC_50_, ½ × IC_50_, and ⅕ × IC_50_. After incubation, the promastigotes were washed twice with PBS and labeled with propidium iodide (PI) (80 μg mL^−1^) for 20 min, followed by centrifugation at 2000× *g* for 10 min and resuspension in 500 μL of PBS. The samples were analyzed using an Accuri C6 flow cytometer (BD Biosciences, Franklin Lakes, NJ, USA) and proprietary software, with at least 50,000 events effectively included in each analysis. All the experiments were performed using biological duplicates in triplicate at each experimental point.

### 3.7. Fluorescence Microscopy

For fluorescence microscopy, assays were carried out with promastigotes of *L. mexicana* in the exponential phase of growth in passage P3 at a concentration of 1 × 10^7^ cells mL^−1^. Treatments were performed with Amp B and the peptides at concentrations of 1.25 or 2.5 μmol L^−1^ for 2 or 24 h, respectively. The parasites were labeled with 5 μg mL^−1^ of Hoechst solution (stock: 1 mg mL^−1^) for 5 min, followed by the addition of PI (1 mg mL^−1^) to a final concentration of 40 μg mL^−1^ and incubation for 20 min at room temperature. Hoechst can permeate through cell membranes, labeling viable cells, and PI is a DNA intercalator that marks cells that have suffered some type of membrane damage. After incubation, the parasites were centrifuged for 10 min at 2000× *g*, followed by resuspension of the precipitate in 100 μL of PBS to remove the fluorophores. The parasites were immobilized on glass coverslips previously treated with Cell-Tak Cell and Tissue Adhesive (Corning™, Glendale, AZ, USA) at 1 μg cm^−2^. Images were acquired with a fluorescence microscope (Nikon DS-R11,10 Nikon Instruments Inc., Melville, NY, USA) at 40× magnification. The experiments were performed using biological duplicates with experimental triplicates.

### 3.8. Inhibitory Activity Against the Enzyme LmCPB2.8ΔCTE (CPB)

The CPB inhibition experiments were performed as described previously by Moreira et al. [50]. The results are expressed as mean ± standard deviation of two independent replicates as the half maximal CPB enzyme inhibitory concentration (IC_50-CPB_) using Bioestat 5.0 software.

## 4. Conclusions

The dimerization strategy was effective for obtaining a compound with leishmanicidal activity and high selectivity. Both the monomer and the dimer showed leishmanicidal activity, with low cytotoxicity and a high selectivity index, but the dimeric peptide was more active and more selective. The dimerization strategy is an effective way to obtain new compounds for the treatment of leishmaniasis, replacing amphotericin B, which lacks selectivity. The degradation assays evidenced that the molecules did suffer degradation, but nonetheless maintained their biological activity.

The permeabilization studies, flow cytometry, and microscopy analyses showed that the likely mechanism of action of the compounds was on the membrane of the parasite. Furthermore, the inhibition of the CPB enzyme by the peptides indicated that at least two mechanisms of action of the compound led to the death of the Leishmania parasite. At concentrations below the IC_50_, the peptides could form transient pores and act on the intracellular targets. At concentrations above the IC_50_, the main target was in the membrane.

These results provide information helpful for the design of new peptides with leishmanicidal activity, showing that the use of dimerization has excellent potential in the discovery of new drugs, offering valuable insights into the development of new therapeutics against *Leishmania*, a significant global public health concern.

## Figures and Tables

**Table 1:** Anti-promastigote and anti-amastigote assays carried out with *L. mexicana*.

Compounds	IC_50_ (µmol L^−1^) Promastigote	IC_50_ (µmol L^−1^) Amastigote 24 h of Treatment	IC_50_ (µmol L^−1^) Amastigote 48 h of Treatment	CC_50_ (µmol L^−1^) Peritoneal Macrophages	SI Amastigote 24 h of Treatment	SI Amastigote 48 h of Treatment
TSHa	6.3 ± 0.85 *	5 ± 0.05 *	8.1 ± 0.45 *	>1000 ± 0.01 *	>200	>123
(TSHa)_2_K	0.6 ± 0.08 *	0.58 ± 0.0 *	0.47 ± 0.08 *	>1000 ± 0.06 *	>1724	>2127
Amp B	0.5 ± 0.2 *	0.63 ± 1.2 *	0.63 ± 1.2 *	24 ± 0.02 *	38	38

* Standard deviation.

**Table 2:** Results of anti-promastigote and anti-amastigote assays carried out with *L. amazonensis*.

Compounds	IC_50_ (µmol L^−1^) Promastigote	CC_50_ (µmol L^−1^) Peritoneal Macrophage	IC_50_ (µmol L^−1^) Amastigote	SI
TSHa	8 ± 0.1	>1000 ± 1.8	6.8 ± 2.82	>294
(TSHa)_2_K	0.6 ± 0.08	>1000 ± 0.35	0.47 ± 0.03	>1724
Amphotericin B	0.7 ± 0.2	24 ± 0.4	0.63 ± 0.02	38

**Table 3:** Results of anti-promastigote assays carried out with *L. amazonensis*, with determination of IC_50_ with active fetal bovine serum.

Compounds	IC_50_ (µmol L^−1^) Promastigote
TSHa	20.0 ± 2.15 *
(TSHa)_2_K	6.12 ± 0.17 *

* Standard deviation.

**Table 4:** The enzymatic inhibition of the CPB enzyme by the TSHA and (TSHa)_2_K peptides.

Peptide	Inhibitory Activity at 20 μmol L^−1^ (%)	IC_50_ (μmol L^−1^)
TSHa	94.22 ± 1.54 *	9.2 ± 2.2
(TSHa)_2_K	98.05 ± 1.02	3.6 ± 0.8

* Standard deviation.

## Data Availability

Data are contained within the article and Supplementary Materials.

## Supplementary Materials

The following supporting information can be downloaded at: https://www.mdpi.com/article/10.3390/molecules29215170/s1, Figure S1: Promastigotes of *L. mexicana* at a concentration of 1 × 10⁷ cells/mL treated with 2.5 μmol L^−1^ of the Amp B for 24 h in (a) cells without marking; (b) *L. mexicana* labeled with 40 μg mL^−1^ of propidium iodide for 5 min; (c) *L. mexicana* labeled 5 μg mL^−1^ of Hoesch for 5 min; (d) Merge of the two markings; Figure S2: Promastigotes of *L. mexicana* at a concentration of 1 × 10⁷ cells/mL treated with 1.25 μmol L^−1^ of the Amp B for 24 h in (a) cells without marking; (b) *L. mexicana* labeled with 40 μg mL^−1^ of propidium iodide for 5 min; (c) *L. mexicana* labeled 5 μg mL^−1^ of Hoesch for 5 min; (d) Merge of the two markings; Figure S3: Promastigotes of *L. mexicana* at a concentration of 1 × 10⁷ cells/mL treated with 2.5 μmol L^−1^ of the Amp B for 2 h in (a) cells without marking; (b) *L. mexicana* labeled with 40 μg mL^−1^ of propidium iodide for 5 min; (c) *L. mexicana* labeled 5 μg mL^−1^ of Hoesch for 5 min; (d) Merge of the two markings; Figure S4: Promastigotes of *L. mexicana* at a concentration of 1 × 10⁷ cells/mL treated with 2.5 μmol L^−1^ of the peptide TSHa for 24 h in (a) cells without marking; (b) *L. mexicana* labeled with 40 μg mL^−1^ of propidium iodide for 5 min; (c) *L. mexicana* labeled 5 μg mL^−1^ of Hoesch for 5 min; (d) Merge of the two markings; Figure S5: Promastigotes of *L. mexicana* at a concentration of 1 × 10⁷ cells/mL treated with 1.25 μmol L^−1^ of the peptide TSHa for 24 h in (a) cells without marking; (b) *L. mexicana* labeled with 40 μg mL^−1^ of propidium iodide for 5 min; (c) *L. mexicana* labeled 5 μg mL^−1^ of Hoesch for 5 min; (d) Merge of the two markings; Figure S6: Promastigotes of *L. mexicana* at a concentration of 1 × 10⁷ cells/mL treated with 2.5 μmol L^−1^ of the peptide TSHa for 2 h in (a) cells without marking; (b) *L. mexicana* labeled with 40 μg mL^−1^ of propidium iodide for 5 min; (c) *L. mexicana* labeled 5 μg mL^−1^ of Hoesch for 5 min; (d) Merge of the two markings; Figure S7: Promastigotes of *L. mexicana* at a concentration of 1 × 10⁷ cells/mL treated with 1.25 μmol L^−1^ of the peptide TSHa for 2 h in (a) Cells without marking; (b) *L. mexicana* labeled with 40 μg mL^−1^ of propidium iodide for 5 min; (c) *L. mexicana* labeled 5 μg mL^−1^ of Hoesch for 5 min; (d) Merge of the two markings; Figure S8: Promastigotes of *L. mexicana* at a concentration of 1 × 10⁷ cells/mL treated with 1.25 μmol L^−1^ of the peptide (TSHa)₂K for 24 h in (a) Cells without marking; (b) *L. mexicana* labeled with 40 μg mL^−1^ of propidium iodide for 5 min; (c) *L. mexicana* labeled 5 μg mL^−1^ of Hoesch for 5 min; (d) Merge of the two markings. Figure S9: Promastigotes of *L. mexicana* at a concentration of 1 × 10⁷ cells/mL treated with 2.5 μmol L^−1^ of the peptide (TSHa)₂K for 2 h in (a) cells without marking; (b) *L. mexicana* labeled with 40 μg mL^−1^ of propidium iodide for 5 min; (c) *L. mexicana* labeled 5 μg mL^−1^ of Hoesch for 5 min; (d) Merge of the two markings; Figure S10: Promastigotes of *L. mexicana* at a concentration of 1 × 10⁷ cells/mL treated with 1.25 μmol L^−1^ of the peptide (TSHa)₂K for 2 h in (a) cells without marking; (b) *L. mexicana* labeled with 40 μg mL^−1^ of propidium iodide for 5 min; (c) *L. mexicana* labeled 5 μg mL^−1^ of Hoesch for 5 min; (d) Merge of the two markings.
