# Supplementary material for: Peptide Dimerization as a Strategy for the Development of Antileishmanial Compounds
Source: Molecules. 2024 Oct 31;29(21):5170. doi: 10.3390/molecules29215170 (PMC11547375; doi:10.3390/molecules29215170)
Supplement: Supplementary file 1 [file molecules-29-05170-s001.zip › molecules-3274787-supplementary.pdf]

Peptide Dimerization as a Strategy for the Development of Antileishmanial Compounds

Natália C. S. Coelho [1], Deivys L. F. Portuondo [1], Jhonatan Lima [1], Angela M. A. Velasquez [1], Valéria Valente [1], Iracilda Z. Carlos [1], Eduardo M. Cilli [2,*] and Márcia A. S. Graminha [1,*]

[1] Department of Clinical Analysis, School of Pharmaceutical Sciences, São Paulo State University (UNESP), Araraquara, SP 14800-903, Brazil; natalia.costa@unesp.br (N.C.S.C.); deivysleandro@gmail.com (D.L.F.P.); jhonatan.lima@unesp.br (J.L.); a.velasquez@unesp.br (Â.M.A.V.); valeria.valente@unesp.br (V.V.); iracilda.zeppone@unesp.br (I.Z.C.)

[2] Department of Biochemistry and Organic Chemistry, Institute of Chemistry, São Paulo State University (UNESP), Araraquara, SP 14800-060, Brazil

* Correspondence: eduardo.cilli@unesp.br (E.M.C.); marcia.graminha@unesp.br (M.A.S.G.)

Support information

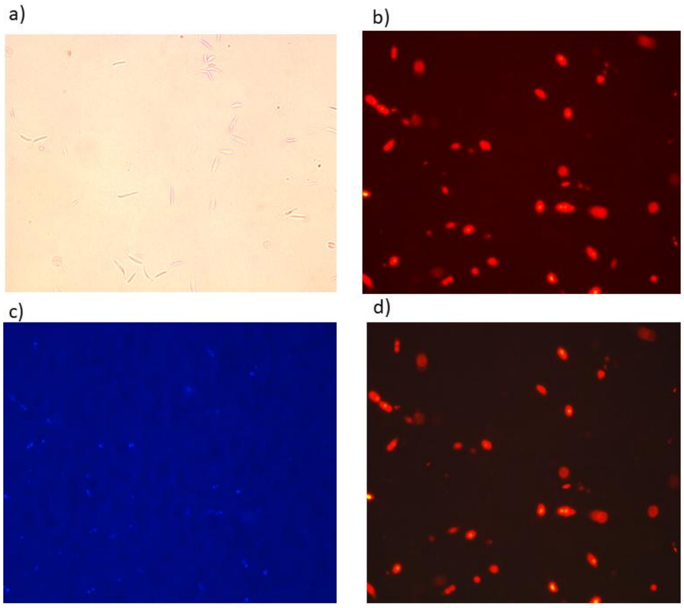

Figure S1: Promastigotes of *L. mexicana* at a concentration of 1x10^7 cells/mL treated with 2.5 µmol L^{-1} of the Amp B for 24 h in a) cells without marking; b) *L. mexicana* labeled with 40 µg mL^{-1} of propidium iodide for 5 min; c) *L. mexicana* labeled 5 µg mL^{-1} of Hoesch for 5 min; d) Merge of the two markings.

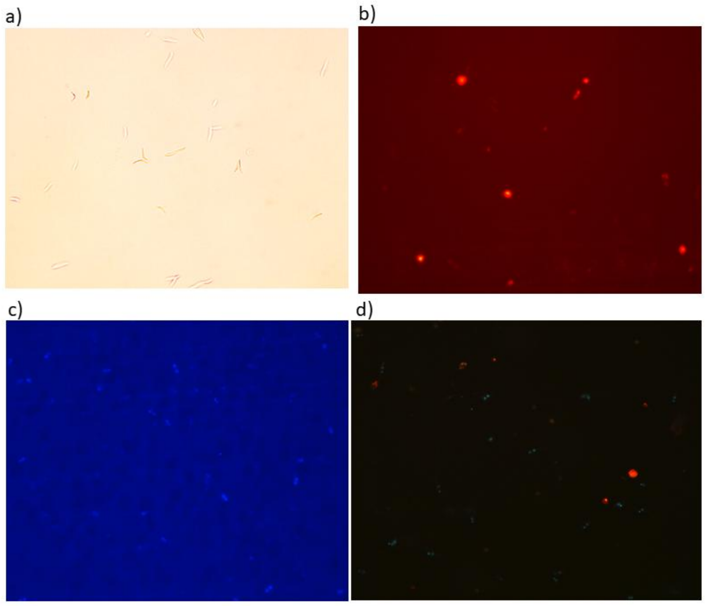

Figure S2: Promastigotes of *L. mexicana* at a concentration of $1x10^7$ cells/mL treated with 1.25 µmol L^{-1} of the Amp B for 24 h in a) cells without marking; b) *L. mexicana* labeled with 40 µg mL^{-1} of propidium iodide for 5 min; c) *L. mexicana* labeled 5 µg mL^{-1} of Hoesch for 5 min; d) Merge of the two markings.

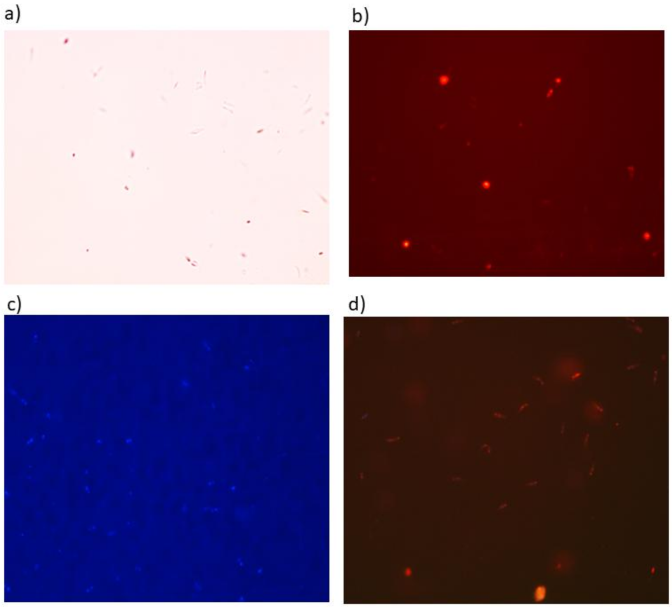

Figure S3: Promastigotes of *L. mexicana* at a concentration of $1x10^7$ cells/mL treated with 2.5 µmol L^{-1} of the Amp B for 2 h in a) cells without marking; b) *L. mexicana* labeled with 40 µg mL^{-1} of propidium iodide for 5 min; c) *L. mexicana* labeled 5 µg mL^{-1} of Hoesch for 5 min; d) Merge of the two markings.

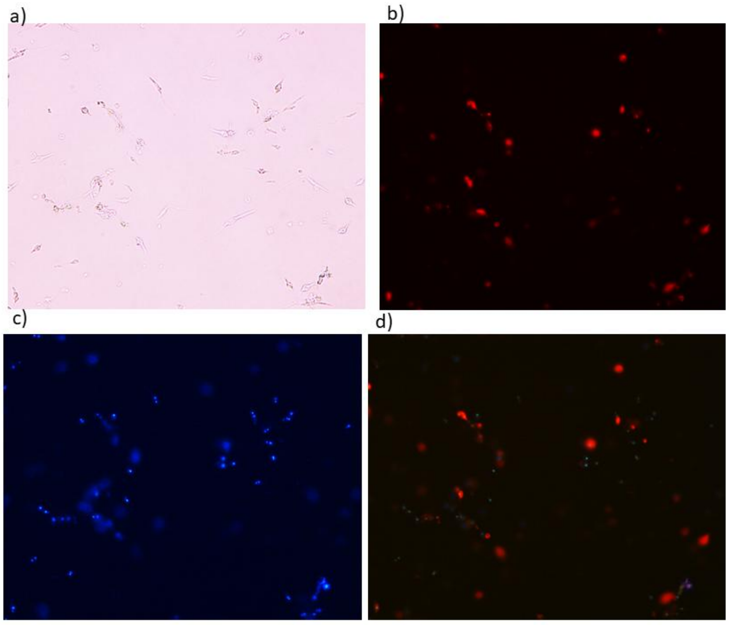

Figure S4: Promastigotes of *L. mexicana* at a concentration of $1x10^7$ cells/mL treated with 2.5 µmol L^{-1} of the peptide TSHa for 24 h in a) cells without marking; b) *L. mexicana* labeled with 40 µg mL^{-1} of propidium iodide for 5 min; c) *L. mexicana* labeled 5 µg mL^{-1} of Hoesch for 5 min; d) Merge of the two markings.

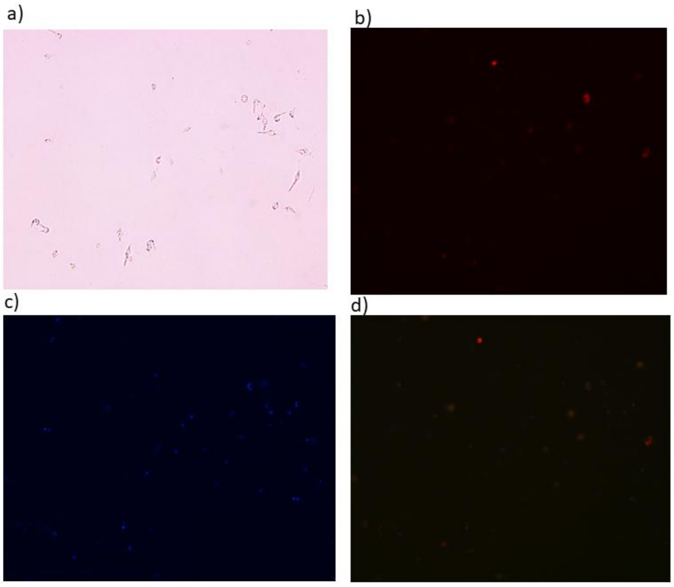

Figure S5: Promastigotes of *L. mexicana* at a concentration of $1x10^7$ cells/mL treated with 1.25 µmol L^{-1} of the peptide TSHa for 24 h in a) cells without marking; b) *L. mexicana* labeled with 40 µg mL^{-1} of propidium iodide for 5 min; c) *L. mexicana* labeled 5 µg mL^{-1} of Hoesch for 5 min; d) Merge of the two markings.

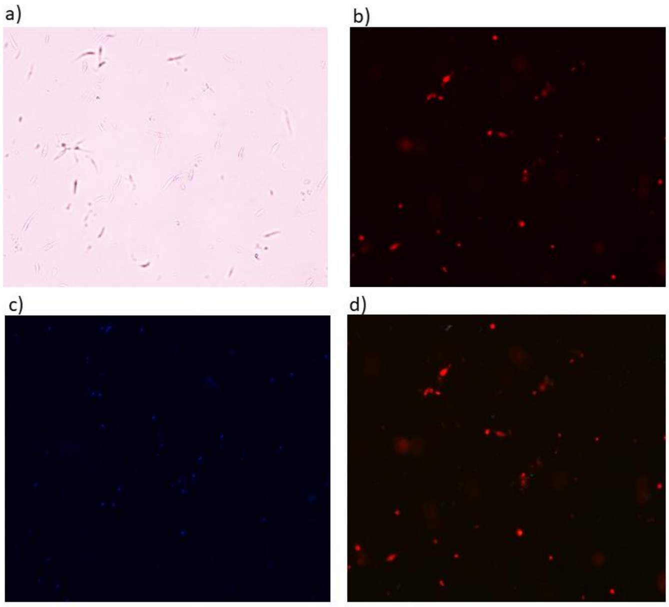

Figure S6: Promastigotes of *L. mexicana* at a concentration of 1x10^7 cells/mL treated with 2.5 µmol L^{-1} of the peptide TSHa for 2 h in a) cells without marking; b) *L. mexicana* labeled with 40 µg mL^{-1} of propidium iodide for 5 min; c) *L. mexicana* labeled 5 µg mL^{-1} of Hoesch for 5 min; d) Merge of the two markings.

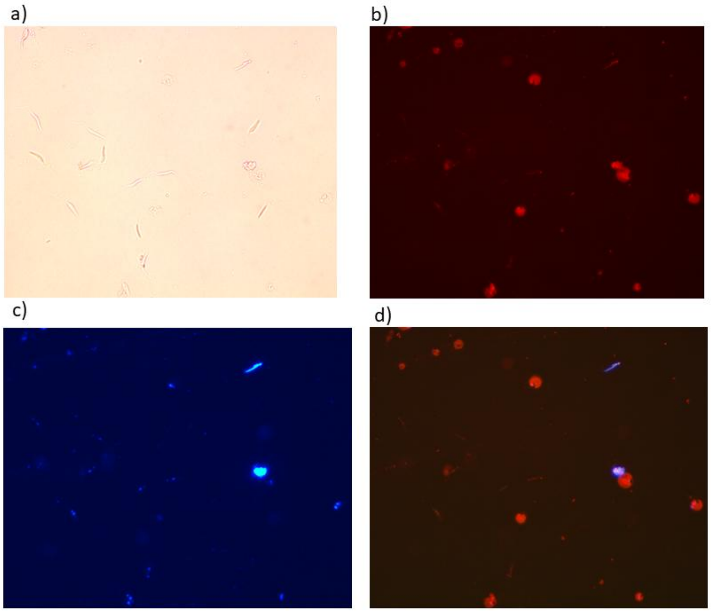

Figure S7: Promastigotes of *L. mexicana* at a concentration of 1x10^7 cells/mL treated with 1.25 µmol L^{-1} of the peptide TSHa for 2 h in a) Cells without marking; b) *L. mexicana* labeled with 40 µg mL^{-1} of propidium iodide for 5 min; c) *L. mexicana* labeled 5 µg mL^{-1} of Hoesch for 5 min; d) Merge of the two markings.

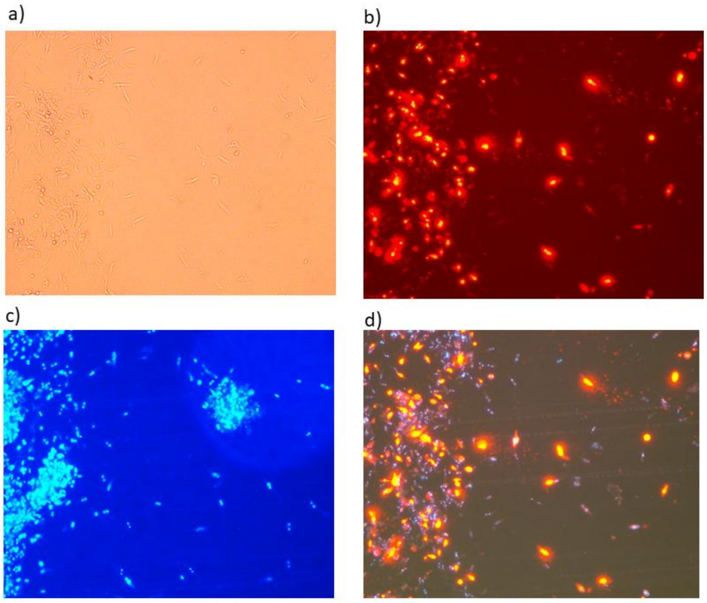

Figure S8: Promastigotes of *L. mexicana* at a concentration of $1x10^7$ cells/mL treated with 1.25 µmol L^{-1} of the peptide $(TSHa)_2K$ for 24 h in a) Cells without marking; b) *L. mexicana* labeled with 40 µg mL^{-1} of propidium iodide for 5 min; c) *L. mexicana* labeled 5 µg mL^{-1} of Hoesch for 5 min; d) Merge of the two markings.

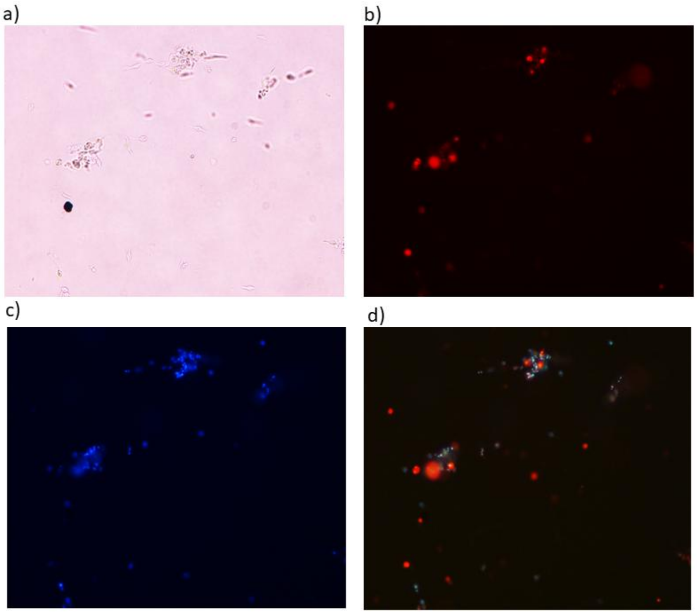

Figure S9: Promastigotes of *L. mexicana* at a concentration of $1x10^7$ cells/mL treated with 2.5 µmol L^{-1} of the peptide $(TSHa)_2K$ for 2 h in a) cells without marking; b) *L. mexicana* labeled with 40 µg mL^{-1} of propidium iodide for 5 min; c) *L. mexicana* labeled 5 µg mL^{-1} of Hoesch for 5 min; d) Merge of the two markings.

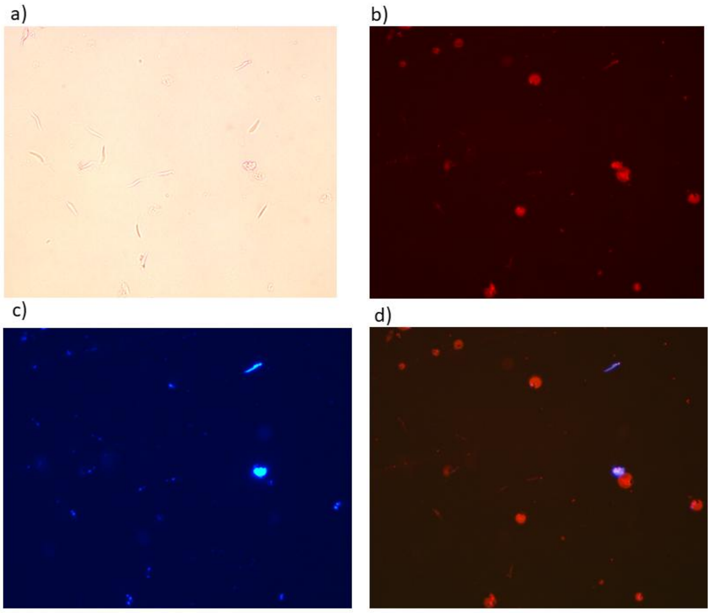

Figure S10: Promastigotes of *L. mexicana* at a concentration of $1x10^7$ cells/mL treated with 1.25 µmol L^{-1} of the peptide $(TSHa)_2K$ for 2 h in a) cells without marking; b) *L. mexicana* labeled with 40 µg mL^{-1} of propidium iodide for 5 min; c) *L. mexicana* labeled 5 µg mL^{-1} of Hoesch for 5 min; d) Merge of the two markings.
